# Grammatical structures of emoji in Japanese-language text conversations

**DOI:** 10.1186/s41235-024-00571-9

**Published:** 2024-07-29

**Authors:** Kazuki Sekine, Manaka Ikuta

**Affiliations:** Faculty of Human Sciences, Waseda University, 2-579-15 Mikajima, Tokorozawa, Saitama 359-1164 Japan

**Keywords:** Emoji, Grammar, Multimodal communication, Japanese

## Abstract

Emojis have become a ubiquitous part of everyday text communication worldwide. Cohn et al. (Cognit Res Princ Implic 4(1):1–18, 2019) studied the grammatical structure of emoji usage among English speakers and found a correlation between the sequence of emojis used and English word order, tending towards an subject–verb–object (SVO) sequence. However, it remains unclear whether emoji usage follows a universal grammar or whether it is influenced by native language grammar. Therefore, this study explored the potential influence of Japanese grammar on emoji usage by Japanese speakers. Twenty adults, all native Japanese speakers, participated in pairs. In Experiment 1, participants engaged in conversations through Google Hangouts on iPads. The experiment consisted of four conversation rounds of approximately 8 min each. The first two rounds involved one participant using only written Japanese and the other using only emojis and punctuation, with roles reversed in the second round. The third round required both participants to use only emojis and punctuation. The results indicated that participants preferred subject–object–verb (SOV) or object–verb (OV) sequences, with OV patterns being more common. This pattern reflects a distinctive attribute of Japanese grammatical structure, marked by the frequent omission of the subject. Experiment 2 substituted emojis for words, showing nouns were more commonly replaced than verbs due to the difficulty in conveying complex meanings. Reduced subject replacements again emphasised Japanese grammatical structure. In essence, emoji usage reflects native language structures, but complexities are challenging to convey, resulting in simplified sequences. This study offers insights for enhancing emoji-based communication and interface design, with implications for translation and broader communication.

**Supplementary Information:**

The online version contains supplementary material available at 10.1186/s41235-024-00571-9.

## Background

Emojis are a type of communication tool widely used around the world. First launched in Japan in 1999, emoji have come into widespread use in tandem with that of cellphones (Yamaguchi & Fujita, 2019). By 2010, smartphones had come into common use and emoji, by then supported by Unicode 6.0, were being widely used not only in Japan but around the world (Takahashi et al., 2021). Unicode is a type of code used to handle characters on a computer in text format rather than as graphic data. It has superior compatibility among computers in various language environments around the world (Nagata, 2020). Emojis include facial emoticons like  or  as well as foods, vehicles, buildings, flags, and symbols. Their wide range of expressions plays an important role in computer-mediated communication (CMC) including communication by social networking services (SNS). According to Yamaguchi and Fujita (2019), the number of emoji has been increasing yearly, with some 82 types of face emoji alone. Each of these highly diverse emoji types plays a different role in CMC. Users select the emoji suited to the roles they need for communication.

Emoji play certain roles of keeping CMC moving smoothly by conveying users’ emotional or semantic messages that compensate for its lack of nonverbal cues in CMC (Takahashi et al., 2021). According to Oyama (2015), the roles of emoji can be divided into three types; *quasi-text*, *illustration*, and *nonverbal complement*. Emoji as a *quasi-text* is used when the relevant sentence is incomplete unless the emoji are converted into letters of some kind. For example, in a sentence like “yesterday, I  and saw a movie with my brother”, the knife-fork emoji is used as an alternative of a text “eat (ate)”. Emoji is used as an *illustration* by adding an emoji related to the semantic content of the sentence. In this way, emoji can iconically illustrate the part of sentence. For example, in a sentence like “yesterday, I had dinner  and saw a movie with my brother”, the emoji added the illustration of “dinner”. Emoji is used as a *nonverbal complement* by providing supplementary information which cannot be gleaned from the text alone, such as “Good to hear  .” In this usage, emojis make invisible facial expressions or inner feelings of the sender visible to the recipient and make it possible to convey playfulness and humour (Kelly & Watts, 2015). Recent studies have demonstrated that facial expression emojis can intensify the emotional valence of text messages, particularly when the emotion of the text message and the displayed emojis are congruent (Boutet et al., 2021; Hand et al., 2022). Just like the nonverbal complement, it has also been suggested that the use of emoji at the end of a sentence can serve to clarify tone of the sentence (Na'aman et al., 2017). By the emoji at the end of the sentence, the recipient can seize the sender’s intention or feeling of the message.

Emojis play a variety of roles in CMC as mentioned above. These roles imply that people may change their interpretations of emoji based on how they relate emojis to the text. For example, the  emoji (hands with palms together) is used not only to mean “thank you” or “please” in a context but also as “praying” or “high five” in other contexts (Naito & Yabuki, 2020). Thus, how a single emoji can take on various meanings depends on the text and context in which the emoji was used. This suggests that the function and interpretation of emoji changes according to the user’s background, especially the language that the users use. In fact, previous research has shown the unique relationship between native languages that are used by emoji users and their interpretations of emoji.

Naito and Yabuki (2020) addressed the difference in the language of texts in which emoji are used to see whether the same emoji can have varying meanings depending on the language used. They examined the difference in emoji meanings between English and Japanese by using data obtained from Twitter. The results showed that in Japanese messages, emojis were often used as an *illustration* to complement text content (e.g. “I went out to lunch  ”), while in English messages, they tended to be used as a *quasi-text* to replace words (e.g. “I had  for lunch”). Naito and Yabuki ascribed the difference of emoji usage to the differing grammar including the word order and grammatical elements that the language users tend to put weight on.

For the word order, most of the currently recognised languages use either S (subject) V (verb) O (object) word order or S (subject) O (object) V (verb) word order (Marno et al., 2015). For example, English is known as a SVO language and Japanese is known as a SOV language. Some studies have examined the relationship between the word order of the language and the gestural communication (Gershoff-Stowe and Goldin-Meadow, 2002; Goldin-Meadow et al., 2008; Langus & Nespor, 2010). Gershoff-Stowe and Goldin-Meadow (2002) conducted an experiment with native English speakers by asking them to describe a short animation, consisting of a couple of objects and an action, to another by using gestures only. The results showed that the order of semantic roles depicted by gestures did not follow the English word order (SVO), and it rather followed the SOV order: agent(S)–patient(O)–act(V). Similar results were obtained in subsequent studies utilising different languages such as Italian and Turkish (e.g. Goldin-Meadow et al., 2008; Langus & Nespor, 2010). Goldin-Meadow et al. (2008) conducted an experiment targeting native speakers of Turkish, Spanish, English, and Chinese, in which they communicated the contents displayed on a computer to a partner who could not see them, using only gestures. The results showed that the gesture order was SOV even for native speakers of SVO languages, thus not dependent on the native language word order.

These findings from previous studies suggest that SOV might be the default order in human languages. However, a study by Marno et al. (2015) reported that this is not always the case. They taught native speakers of Italian (SVO) and Persian (SOV) a set of gestures and later asked them to describe simple events by using only the gestures. The results showed that the gesture strings were more likely to follow an SVO order if participants were given the known gesture vocabulary. However, the gesture strings followed SOV order when participants used improvised gestures. Thus, this study suggested that when a gestural lexicon is given to participants, SVO emerges.

In daily communication, we convey messages by using not only gestures and speech but also emojis. On the one hand, emojis resemble gestures in that they both iconically convey meanings, but on the other hand, emojis are similar to language as they both use a set of ready-made symbols. If we use only a sequence of emoji to convey a message, does the order of emoji depend on the user’s native language word order? Cohn et al. (2019) conducted an experiment having English speakers converse online using only emoji, in order to examine whether there was a relationship between emoji usage methods and grammatical structure. They found that emoji were mostly used in SVO order, influenced by English word order. They also showed that the grammatical structures tended towards the simple, because conveying complex information by only emoji is difficult. Further, in one of four experiments, they asked participants to replace sentences they made with emoji. The result was that the participants tended to replace nouns with emoji more often than verbs. From these findings, however, it is difficult to disentangle whether participants followed their native language word order or SVO word order for the emoji strings because the experiments were conducted solely in English, an SVO language. Furthermore, although it was reported that data were collected from participants with various language backgrounds, the participants' first languages and their English proficiency levels were not specified. This diversity in language background could complicate the results, making it challenging to determine what influences the order of emoji strings. Therefore, to understand the influence of language, it is preferable to focus on monolingual speakers. It is also important to examine whether the emoji strings follow an SVO order when participants use the known emoji vocabulary, just like Marno et al.’s (2015) finding. To address these issues, we targeted monolingual native speakers of Japanese, which is an SOV language, in the current study.

By using picture cards, Takano and Nakata (2013) carried out an experiment with native Japanese and English speakers based on the assumption that the use of picture cards would be more linguistic than the "silent gestures" (gestures without speech) used in Goldin-Meadow et al.’s (2008) study. The results showed that, unlike silent gestures, the order of picture cards was dependent on the native language word order, influenced by the native language. Thus, when the set of static symbols was given to the users in advance and the users have to convey messages by using it, they tend to depend on the native language word order. If emoji are also treated like the picture cards, the order of emojis would follow the native language word order, rather than the SVO order found by Cohn et al. (2019).

Thus, the current study investigated whether the emoji order of native Japanese people followed Japanese grammar or the SOV order, by following up Cohn et al.’s (2019) study. By targeting monolingual speakers and utilising the same task and procedure as those used in their study, we were able to focus on the influence of language on the order of emojis. To this end, we conducted two experiments. In Experiment 1, we examined how Japanese grammar influences emoji usage methods by prompting conversation using only emoji and punctuation. In Experiment 2, we scrutinised the interactions of emoji with textual semantic and grammatical structures by having the participants deliberately replace text messages that they had already created themselves, with emoji.

For Experiment 1, we predicted that conversation using emoji is influenced by Japanese grammar and thus follows SOV word order, as Takano and Nakata (2013) study found with picture cards. This is because emoji and cards seem to be similar in that a set of emoji or cards is known in advance by participants. For Experiment 2, we expected that components other than the subject would be frequently replaced by emoji, because it is likely that subjects are often omitted in Japanese text, as Kato (2012) points out.

## Experiment 1

The purpose of experiment 1 was to examine how Japanese grammar influences the order of emoji by prompting conversation using only emoji and punctuation.

### Experiment design

The experiment used a three-factor within-participant design including conversations in which one of the pair used only emoji and punctuation and in which both of the pair used emoji and punctuation.

### Participants

Twenty native monolingual Japanese-speaking adults (9 males, 11 females, average age 20.45, SD = 0.67, range = 19–22) participated in the experiment. They participated as a pair and all the pairs were composed of friends. According to the post-experiment questionnaire, 28.6% of the participants had experience using the chat application (Google Hangouts) used in the experiment.

### Procedures

We used same procedure as Cohn et al.’s (2019) study as we aimed to follow up their study with Japanese speakers. The experiment was conducted individually in the university laboratory.

First, a pair of participants entered an experimental room, and then they were explained that they were taking part in an experiment on university students’ emoji usage methods. Then, to familiarise them with conversation on iPad (apple) using the Google Hangouts software application, they were given time to use the tablet to conduct short conversations in words and in emoji. This application was chosen because the conversations can be exported for later analysis, and it could be used without requiring a phone number. After they practiced how to use the application, each participant was placed in the opposing corners of the room and asked to face the wall so that they could not see each other. They were also asked not to speak out loud during the experiment. An experiment consisted of four different rounds of conversation, each with a different topic and restriction of emoji use. Each round lasted about eight minutes.

By following Cohn et al.’s study, the tasks for the first three rounds of the experimental session were called Experiment 1 and the fourth round was called Experiment 2. In the first round of conversation, one participant was allowed to use written Japanese words, while their partner was asked to communicate using only emoji and punctuation (including “!” and “?”). In the second round, the participants were asked to switch roles, reversing the usage employed in the first round. In both rounds, there was no restriction placed on the use of emoji to supplement the conversation by the person using written Japanese words. In the third round, both participants were asked to communicate with emoji and punctuation only, without written words. Emoji containing text, such as  , were also acceptable. In the fourth round, which is Experiment 2, participants were asked to communicate while replacing part of the message with emoji. The results of this task are analysed in Experiment 2.

To facilitate conversations between participants, we provided a conversational prompt with topics of discussion. In accordance with the study by Cohn et al. (2019), the topics selected were "the perfect date", "travelling", "future plans", and "zoo visit". They selected these topics based on categories of emoji used in the Google emoji lexicon in 2016, according to emojipedia.org. To encourage communication between participants, leading questions were prepared for each topic, such as “Ask the other person to help you plan the perfect date”, “Get the other person to tell you about their future plans over the next ten years”, etc.

In addition, guiding questions, such as such as “What would be a perfect date for you?” and “How would you describe your perfect partner?” were also provided. The order of the topics used in conversation was randomly assigned for each round in a way that across all participants, each topic was used an equal number of times in each experimental round. After completing all the experiments, participants were individually asked by the experimenter to describe the meaning of the emoji strings they had created during the experiments. The experiment lasted from 30 to 60 min in total.

### Ethical considerations

Participants were informed that the names of individuals would not be released and the data would be anonymously analysed. They also received an explanation of the study and filled in an informed consent. After each test, the conversations that each pair had made were exported from the tablet to a computer to save them, without their personally identifiable information. Then, their conversation histories were cleared from the Google Hangouts before the next test started.

### Emoji coding

The current study analysed "segment" as a unit of analysis in the annotation. A segment was defined by Cohn et al. (2019) as any span of emoji that formed a recognisable grouping. By following their procedure, we first identified an isolated line of conversation set apart by a message break, or, less frequently, as multiple segments within a single line of conversation. These segments were determined first by consulting participants’ own annotations and second by our analytical categories. It is important to note that annotations were not mutually exclusive, and that a segment could be categorised into multiple categories, if needed.

We then classified emoji segments into 24 categories. Cohn et al. (2019) used 23 categories for emoji grammar based on hierarchy of grammatical complexity suggested by Jackendoff and Wittenberg’s (2014) study. We added one more category called “single-word responses” to them, because the Japanese language, as a pro-drop language, involves a high frequency of conversation using single words alone. This was also true for a conversation with emoji. The definition and examples of each Emoji category are indicated in Table 1.

**Table 1:** Emoji categories in segments composed of emoji alone

Emoji type	Definition	Example
*One-unit grammars*		
Formulaic expressions	Emoji serving conversational functions (yes, no, hmm)	
Responsive emotions	Emoji used for feelings or emotions	
Single-word responses	Expression with only one word	: go by train, : eat curry
*Linear grammars*		
Temporal sequence	A linear sequence of events	: go see rabbits, koalas, and pandas in that order
Unrelated list	List of emoji with no intrinsic semantic relationships	: get in the car and go to the beach and eat dinner
Semantic list	Emoji related by a semantic associative field	
Reduplication	Repetition of the same emoji	: like very much
*Categorical grammars*		
Three-unit (SVO, SOV, etc.)	Three-unit sequence of emoji playing “grammatical” roles	: I drive to the amusement park
Two-unit (SO, SV, VS, etc.)	Two-unit sequence of emoji playing “grammatical” roles	: travel to Italy
*Simple phrase grammars*		
Embedded sequencing	Sequencing where one grouping was embedded in another grouping	: A couple drives to a beautiful sea full of fish
*Other classifications*		
Metonymy	An emoji with a related meaning to the actual message	: work/job
Rebus	Use of an emoji for its phonological correspondence unconnected to its visual meaning	: go back home
Affixation	Attachment of two emoji to create a larger single unit	: beef
Whole image	Combination of emoji to create a single “picture”	: family trip

The 24 categories have four upper categories; “one-unit grammars” “linear grammars,” “categorical grammars,” and “simple phrase grammars”, which are explained below.

The most basic emoji usage is called "one-unit grammars" where sentences are composed of a single word. This upper category includes three subcategories. Use of emoji that are close to *formulaic expressions* include usages expressing emotions (serving conversational functions), such as “Yes:  ” and “No:  ”. *Responsive emotions* are emoji used to express emotion in response to the partner, either alone, as in  , or in combination with other emoji, as in  . Cohn et al. (2019) included only these two classifications under "one-unit grammar". But because emoji expressions using only a single word were frequently observed in the current study, the “single-word response” was added under this category. Examples include “  ” (a hamburger) in response to “What do you want to eat?” or “  ” (by train) in response to “How do you get there?”.

The next upper category is called "linear grammars", which refer to strings of words connected by semantic relations. There are four subcategories in "linear grammars". *Reduplication* refers to the repeated use of the same emoji, often used for emphasis, as in “  ,” liking someone or something a lot. *Semantic list* is a combination of common semantic associative emoji, as in “  ,” which indicates a list of work. *Unrelated list* is a combination of emoji that are not semantically unified but are related to the context in the conversation. “  ” is an example, which means going to see cherry blossoms at lunchtime. *Temporal sequence* conveys sequential events in chronological order. In a temporal sequence, the order of the emoji is important in order to understand the meaning.

The next upper category is called "categorical grammars", which refers to sequences where "units had a clear word-like order where emoji played relative roles as belonging to categorical grammars. These sequences could be either *three-units* long (SVO, SOV, etc.) or *two-units* long (SV, SO, OS, OV, VS, VO)" (Cohn et al., 2019, p. 7). By following Cohn et al.’s study, we collapsed the semantic notions of agents and sources to “subjects”, patients and goals to “objects”, and actions to “verbs”.

"Simple phrase grammars" is another upper category, which contains one subcategory, called *Embedded sequencing*. Embedded sequencing represents that one grouping of emoji is embedded within a larger sequence. For example, “  ” contains a term for a happy couple within a sentence about going to an amusement park by car to ride the ferris wheel. The embedded content may also belong to other categories, such as a semantic list or affixation.

The last upper category is called "other classifications", which has four subcategories. Emoji segments that were not fallen into classification of grammatical levels were fallen into this category. *Metonymy* delivers a meaning made by a combination of mutually related emoji, such as “  ” depicting “cooking”. The emoji represent more abstract concepts or words than what each emoji itself represents. *Rebus* is the emoji where the sound quality of an emoji is used to represent a word as in “  ” to mean “returning home” in the Japanese context; the word "frog" in Japanese is "kaeru", which sounds same as the word "returning". *Affixation* indicates the creation of a word with more detailed meaning, by combining emoji, retaining the appearance of a single image. For example, this smoke emoji “  ” can be affixed to a person running or a car, as in “  ” or “  ,” to produce a sense of speed. Affixation emoji play the role of modifying words. *Whole image* also represents a single iconic image-unit but without affixation, as in “  ” for a couple in a car or “  ” for a person eating pizza.

The current study used "one-unit grammars", "linear grammars", "categorical grammars", and "simple phrase grammars" as its four upper categories, with 24 subcategories.

### Data analysis

We analysed all emoji-only segments produced from rounds 1 to 3 in Experiment 1.

First, of the target segments, we calculated how many times each category was used for each participant. Note that a segment was sometimes classified into multiple categories. Next, the proportion of each category was calculated by dividing occurrence of each subcategory by the total number of occurrences in all categories. To understand whether certain patterns were used at a significant rate overall, using one-sample *t* tests, we compared the means for each category against the frequency rate of 0.042—the chance of a category occurring 1 time out of the 24 total categories analysed (Cohn et al.’s (2019) study used 23 categories, hence the frequency rate was 0.043). We then analysed categories used at a significantly higher proportion than the chance level. To compare the proportion between categories within an upper category, paired t-tests were used for comparisons between two subcategories alone, while repeated-measures analysis of variance (ANOVA) were used to examine more than two subcategories, followed by post hoc comparisons with a Bonferroni correction.

## Results

A total of 414 segments were analysed. The participants conducted an average of 15.33 conversations (SD = 5.73) per round. The participants used an average of 1.28 segments (SD = 0.34) per turn, with 2.21 emoji per segment. The segments were identified per one message line.

We first analysed the proportion of emoji usage in the upper-level categories of "one-unit grammars", "linear grammars", "categorical grammars", and "simple phrase grammars". Subsequently, the analysis addressed the 24 subcategories.

### Number of emoji and relationship to complexity of grammar

**Fig. 1:**
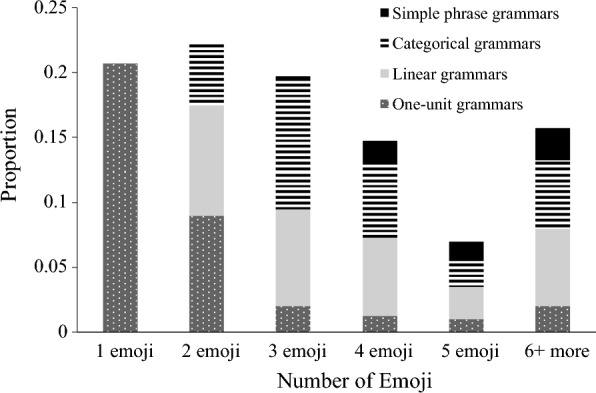
Number of emoji used in emoji-only utterances and the types of sequencing complexity used in those utterances. Note that one-unit grammars could contain more than one emoji

We counted the number of emoji used in emoji-only utterances and then classified each emoji-only utterance into upper-level categories, which indicate types of sequencing complexity used in those utterances. To this end, the proportion of each category was calculated through division by the total number of all categories (Fig. 1). As shown in Fig. 1, there was a relationship between the number of emoji and grammatical complexity: the fewer emoji used, the simpler the grammar was, and likewise in reverse. In addition, sentences using two or more emoji were found in all upper-level categories except "one-unit grammars".

### Comparison among the four upper-level categories

Each participant’s segments composed only of emoji were classified into the four upper-level categories (one-unit grammar, linear grammar, categorical grammar, and simple phrase grammar), and the total of each category was calculated. Next, the proportion of use was calculated by dividing by the total of all categories, and a one-way ANOVA was performed. A main effect of the category was found in the frequency of occurrence, *F* (3, 57) = 28.71, *p* < 0.001, $\eta_{p}^{2}$ = 0.60.

Post-hoc comparisons (Bonferroni, *p* < 0.05) showed that one-unit grammars (*M* = 0.53, SD = 0.06) were used more often than the other categories, followed by categorical grammars (*M* = 0.37, SD = 0.04), linear grammars (*M* = 0.33, SD = 0.05), and single-phrase grammars (*M* = 0.05, SD = 0.02). Except for linear grammars and categorical grammars (*p* = 0.37), a significant difference was found in all combinations (*p* < 0.05).

### Comparison among 24 subcategories

**Fig. 2:**
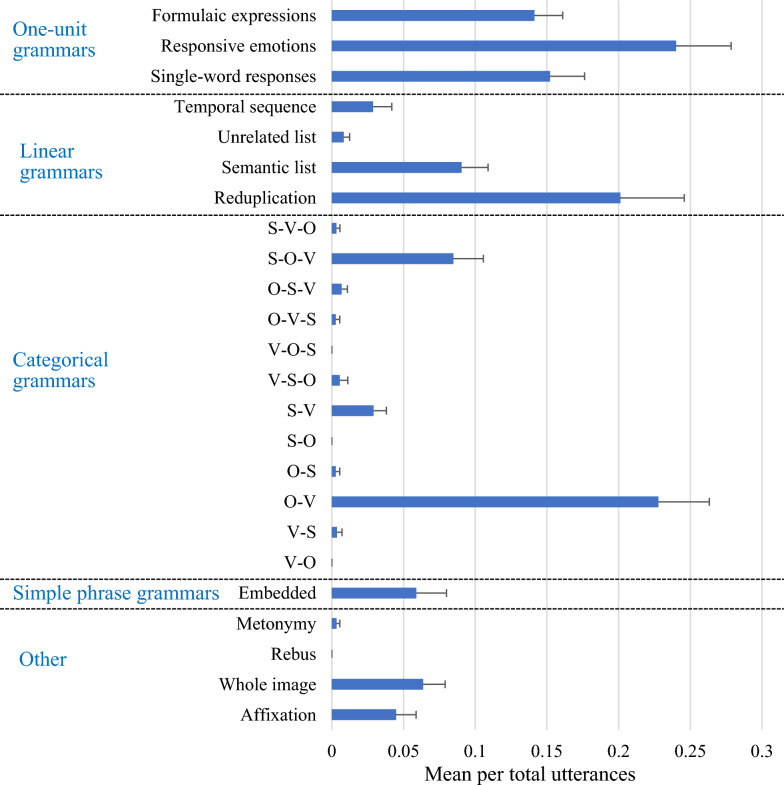
Types of utterances used in emoji-only conversations. Error bars indicate standard error. O objects, S subject, V verb

Each participant’s segments composed only of emoji were classified into 24 subcategories and the total for each category was calculated. The details of data are described in the “Supplementary Materials”. The proportion of use was calculated by dividing by the total of all categories, and a one-way ANOVA was performed. The results found a significant difference in the frequency of occurrence, *F* (23, 460) = 22.25, *p* < 0.001, η_p_^2^ = 0.47. The average proportion of use for each category is shown in Fig. 2.

First, in order to find any differences in frequency of use between categories, one-sample t-tests were used to compare the average proportion of use of each category with the frequency rate of 0.042, the probability that each of the 24 categories would appear once (chance level). The results found significantly high occurrences of formulaic expressions, *t* (19) = 5.05,* p* < 0.001, responsive emotions, *t* (19) = 5.16, *p* < 0.001, single-word responses,* t* (19) = 4.58, *p* < 0.001, semantic lists,* t* (19) = 2.64, *p* = 0.016, reduplication, *t* (19) = 3.58, *p* = 0.002, and object-verb,* t* (19) = 5.23, *p* < 0.001. All the subcategories in the "one-unit grammar" category were used at a rate significantly higher than the chance level. Only reduplication in the linear grammars category and object-verb in the categorical grammars category were significantly higher. There were no subcategories significantly higher than the chance level in "other classifications" and "rebus" was not used at all.

### Proportion of use of each subcategory in "one-unit grammars"

**Fig. 3:**
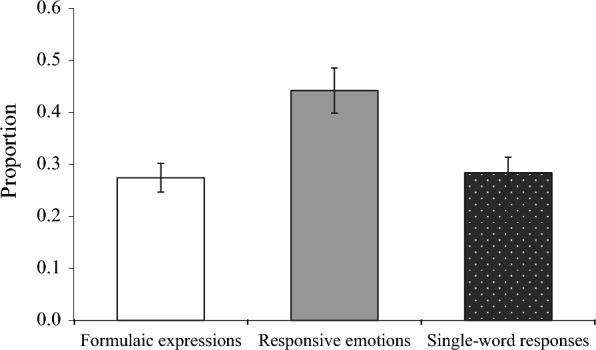
Proportion of use of each subcategory in "one-unit grammars”. Error bars indicate standard errors

In order to find any differences in frequency of use within the "one-unit grammars" category, a one-sample t-test was used to compare the average proportion of use of each subcategory with the frequency rate of 0.33, the probability that each of the three categories would appear once (chance level) (Fig. 3). The results showed that only "responsive emotions" were used at a significantly higher rate than the chance level, *t* (19) = 2.58, *p* = 0.018.

### Proportion of use of each subcategory in "linear grammars"

**Fig. 4:**
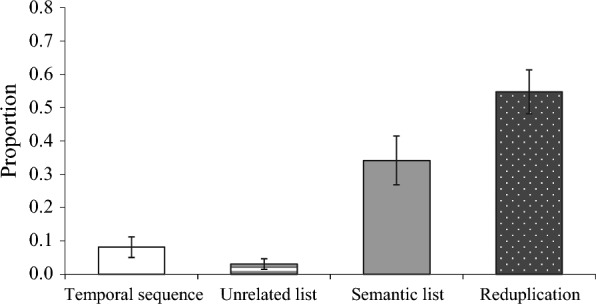
Proportion of use of each subcategory in "linear grammars". Error bars indicate standard errors

To find any differences in frequency of use within the "linear grammars" category, a one-sample t-test was used to compare the average proportion of use of each subcategory with the frequency rate of 0.25, the probability that each of the four categories would appear once (chance level) (Fig. 4). The results showed that only "reduplication" was used at a significantly higher rate than the chance level,* t* (19) = 4.51, *p* < 0.001.

### Proportion of use of each subcategory in "categorical grammars"

**Fig. 5:**
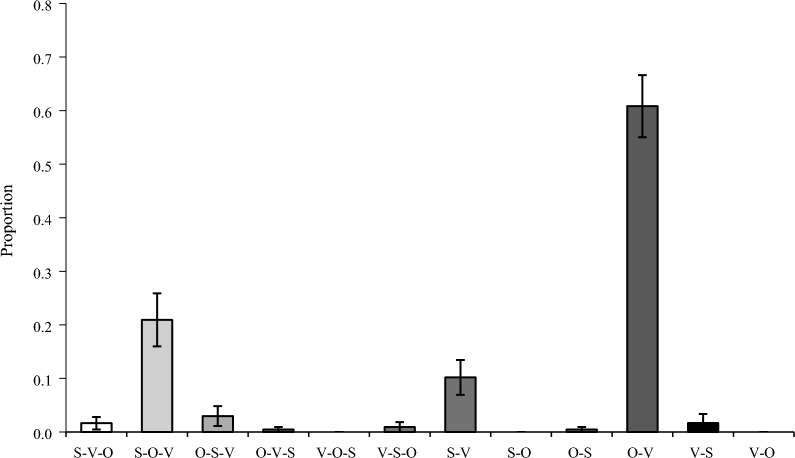
Proportion of use of each subcategory in "categorical grammars". Error bars indicate standard errors

To find any differences in frequency of use within the "categorical grammars" category, a one-sample t-test was used to compare the average proportion of use of each subcategory with the frequency rate of 0.083, the probability that each of the 12 categories would appear once (chance level). The results are shown in Fig. 5. "Subject-object-verb", *t* (19) = 2.55, *p* = 0.020, and "object-verb", *t* (19) = 9.05, *p* < 0.001, were used at significantly higher rates than the chance level. "Subject-verb" was often used as well but it was not significantly higher than the chance level. The usage rate of the SVO word order, considered the common basic word order in communication methods including gestures, sign language, and spoken language, regardless of language type, was 0.02, lower than the chance level. Word orders including VOS, SO, and VO were not used at all.

### Discussion of experiment 1

In experiment 1, the participants were asked to converse using only emoji and punctuation, and the results thereof were classified into grammatical categories. Overall, the participants’ conversation was characterised by the frequent use of "one-unit grammars" and "linear grammars" with simple grammatical structures used. This is in accordance with the trends indicated by Cohn et al. (2019). On the other hand, the second most frequent category was "categorical grammars", with no significant difference from "linear grammars", a finding differing from the results of Cohn et al. (2019). The more frequent use of categorical grammars in this study showed that Japanese use emoji not only as a method of expressing emotions and moods, but also as a method of conveying semantic information in the same way as they do in spoken language. In addition, as shown in Fig. 1, the most common segments were those using two emoji, followed by single-emoji segments. This is another point differing from the experiment conducted by Cohn et al. (2019) with English speakers, which found that single-emoji segments were the most common. A possible explanation, as seen in the frequent use of categorical grammars in this study, is that there were more segments using more complex grammar.

In the one-unit grammars, the most common subcategory was responsive emotions. Based on this point, as shown by Cohn et al. (2019), emoji seem to be used as nonverbal methods of conveying emotions and moods. Furthermore, the "single-word response" added to the study, which was not distinguished by Jackendoff and Wittenberg (2014), was also common. According to Kato (2012), it is more frequent in Japanese than in English for subjects to be omitted. The tendency in Japanese to omit the subject is thought to have produced a large number of segments composed only of single words.

In the linear grammars category, reduplication was the most common, followed by semantic lists. Similar to the tendency found by Cohn et al. (2019), repeated emoji are used to indicate greater emphasis or larger quantities, as in “  ” (very sad), “  ” (a lot of curry), or “  ” (lots of food). Unrelated lists were found in Cohn et al. (2019) with the same frequency as temporal sequences, but here hardly appeared at all. This category was frequently found in segments using emoji with a subject, such as “  ” (I saw a beautiful nightscape). On the other hand, because Japanese tends to omit the subject, as in “  ” (saw a beautiful nightscape), unrelated lists were relatively uncommon even in segments using emoji.

In the categorical grammars category, "object-verb" was the most frequent, followed by "subject-object-verb". The use of these two subcategories was significantly more frequent, which matches Japanese grammatical word order. On the other hand, the "subject-verb-object" frequently seen in Cohn et al. (2019), said to be the basic grammatical order common across communication methods, was rare in this study, lower than the chance frequency rate of 0.083 at which each of the 12 subcategories would appear once. These findings suggest that the normally used native language influences communication using emoji. Moreover, the reason "object-verb" was used more frequently than "subject-verb-object" came from the fact that Japanese is a pro-drop (pronoun-dropping) language, unlike English.

There were no significantly more frequently used subcategories in single-phrase grammar or other classifications. As this indicates, segments composed only of emoji and punctuation tend to use one-unit grammars, linear grammars, or categorical grammars. The fact that rebuses were not used at all suggests that the participants were not using emoji in phonological roles.

The results of Experiment 1 suggest that conversation using emoji is influenced not by universal common grammar regardless of people’s native language, but is affected by the language normally used. The frequent use of emoji in SOV and OV word order and of grammar omitting the subject is thought to be the result of the influence of Japanese grammar and characteristics. Finally, this study found that emoji were used not only as a method of expressing emotions and moods, but also as a method of conveying semantic information in the same way as spoken language.

## Experiment 2

Experiment 2 was conducted to examine the mutual interactions of emoji with the semantic and grammatical structures of sentences. This experiment focused on the mutual interactions when emoji replaced words by having the participants deliberately replace words by emoji in sentences they had created before. By following Cohn et al.’s (2019) study, this experiment prompted replacement with emoji within sentences.

Potter et al. (1986) found that, in a study on the semantic understanding of images within sentences, sentences replaced with images are more difficult to understand than those using text. On the other hand, when only nouns and verbs in sentences were replaced by images, it was found that the sentences are almost as easy to interpret as sentences using text alone. This suggests that accurate interpretation is possible only with sentences with simple grammatical structures, with words frequently used in everyday life replaced, or with only a small range of words or sentences replaced.

Previous research by Cohn (2016) showed that sentences using emoji in place of words take longer to read than sentences composed of text, and may also be misunderstood depending on the context. Also, when nouns and verbs are replaced with emoji, more time is required than when reading sentences composed of text. This finding is also confirmed by a recent study (Paggio & Tse, 2022) using eye-tracking measures. However, Cohn revealed that when similar replacement takes place with the normal grammatical structure (the emoji replacing nouns are in the noun position and those replacing verbs in the verb position), the sentences can be read in less time than those where replacement differs from the normal grammatical structure (the emoji replacing nouns are in the verb position, and/or vice versa). This suggests that emoji have mutual interaction with the grammatical structure of sentences.

Cohn et al. (2019) conducted an experiment in which participants were asked to replace one or more parts of a sentence with pictograms in a conversation, based on the fact that the degree of difficulty in creating and understanding the meaning of a sentence changes depending on the size of the area to be replaced and the grammatical category. The results found that nouns and adjectives were replaced more frequently than verbs and adverbs. In addition, the emoji used tended to be related to the text part of the sentence, with few duplicating text content or being unrelated thereto. This study conducted an experiment similar to Cohn et al.’s (2019) study, with participants who were native speakers of Japanese, which has a different grammatical structure from the language of the participants in Cohn’s experiment, in order to examine whether different interactions of emoji could be found with the semantic and grammatical structure of sentences.

### Experimental design

The experiment used a one-factor within-participant design.

### Participants

The same participants as in experiment 1 took part in experiment 2.

### Procedures

Among the four rounds of the experiment, this fourth round was intended to clarify the mutual interaction of emoji and text in grammar. In this round, both participants were able to use text, but were asked to use at least one emoji to replace characters in their sentences.

### Data coding

This study focused, in accordance with the research of Cohn et al. (2019), on what grammatical category of the sentence was replaced with emoji and on the relation of these emoji with the sentence’s semantic structure. Therefore, first, the position of the replacement emoji relative to the text (within a sentence, at the end of a sentence or substituting for the whole sentence) was classified. When multiple replacement emoji existed within one sentence, the sentence was sometimes classified into multiple categories. Next, the emoji replacing words were classified by grammatical category (subject, object, verb, noun, adjective, adverb, preposition) and semantic category (semantic object, animate object, action, property, location). In addition, the semantic relations of the sentences consisting of emoji and text were categorised by multimodal relations (extratextual, associative, metonymy, redundant). When the meaning of the emoji is included in the text part, it is "overlap", and when it is not mentioned in the text, it is "extratextual". Further, the emoji part was categorized (as in experiment 1) into structural types: one-unit grammars (formulaic expressions, responsive emotions, single-word responses), linear grammars (temporal sequences, unrelated lists, semantic lists, reduplication), and so on.

### Data analysis

This experiment analysed the fourth-round segments for the mutual interactions of emoji with the semantic and grammatical structure of sentences. In this round, the participants were asked to converse using text, and at the same time to replace at least one item in each sentence with an emoji.

First, the replacement emoji were classified by position in the sentence (middle, end, or isolated), and the totals of each category calculated. Replacement within the sentence indicates sentences like “In  (winter), Korea is cold”. Replacement at the end of the sentence indicates sentences like “going to the sea to  (swim)”. Substitution for the whole sentence indicates responding to a question like “What do you want to eat?” with a single  emoji. Then, the results were divided by the total of all categories to calculate the proportion of use for each participant, using a one-sample t-test for comparison with the frequency rate of 0.33, the probability that each of the 3 categories would appear once (chance level).

Next, as in experiment 1, of each participant’s analysis target segments, the total of subcategories within "grammatical categories", "semantic categories", "multimodal relations", and "structural types" was calculated for each participant. The results were then divided by the total of all categories to calculate the proportion of use, using one-sample t-tests to analyse for differences in the frequency of use of each category. Here, the average proportion of use for each category was compared with the probability that each category would appear once (chance level). For example, as there were six subcategories of grammatical categories, each was compared with 1/6 (0.167). This value was 1/5 (0.2) for semantic categories, 1/4 (0.25) for multimodal relations, and 1/6 (0.167) for structural characteristics.

## Results

A total of 229 segments were analysed. Participants used 1.38 emoji (SD = 1.06) per segment. The results of calculating the average proportions of the position of the replacement emoji were that there were significantly fewer the end of the sentence (*M* = 0.10, SD = 0.13) and as substitution for the whole sentence (*M* = 0.01, SD = 0.03), compared to within the sentence (*M* = 0.89, SD = 0.13). To investigate whether a significant proportion of replacements took place at specific positions, a one-sample t-test was used to compare the average proportion of uses of each category with 0.33, the frequency rate if each of the 3 categories appeared once. The replacement emoji within the sentence was significantly higher than the chance level, *t* (19) = 18.65, *p* < 0.001. We did not find any significant differences in the other two replacement positions.

Next, the proportion of use for each subcategory of grammatical categories, semantic categories, multimodal relations, and structural types was calculated per participant. In the grammatical categories (1/6: 0.167 chance), noun replaced with emoji was significantly higher than the chance level, *t* (19) = 16.24, *p* < 0.001, while verb, object, adjective, and adverb all appeared at a significantly lower rate, all *t* (19) < − 31.09, all *p* < 0.01. Prepositions were omitted from the analysis, as they were not replaced.

In the semantic categories (1/5: 0.20 chance), semantic object was significantly higher than the chance level, *t* (19) = 6.17, *p* < 0.001, while location were higher than the chance level 0.2, but with no significant difference (*p* = 0.76). Animate, *t* (19) = − 1.13, *p* = 0.195, action, *t* (19) =  − 4.31, *p* < 0.001, and property, *t* (19) =  − 2.66, *p* = 0.015, were significantly lower than the chance level.

In the multimodal relations (1/4: 0.25 chance), associative, *t* (19) = 0.88, *p* = 0.391, and metonymy,* t* (19) = 1.75, *p* = 0.096, were higher than the chance level 0.25, but with no significant difference, *t* (19) =  − 5.31, *p* < 0.001. The occurrences of redundant, *t* (19) =  − 0.64, *p* < 0.532, and extratextual, *t* (19) =  − 5.31, *p* < 0.001, were significantly lower than the chance level.

In the structural types (1/6: 0.167 chance), semantic lists were significantly higher than the chance level, *t* (19) = 2.96, *p* = 0.009, while responsive emotions were higher than the threshold value of 0.167 but with no significant difference, *t* (19) = 1.52, *p* = 0.144. Temporal sequences, *t* (19) =  − 19.40, *p* < 0.001, unrelated list, *t* (19) =  − 1.58, *p* = 0.130, formulative expression, *t* (19) =  − 2.34, *p* = 0.030, reduplication, *t* (19) =  − 1.90, *p* = 0.072, were significantly lower than the chance level.

### Discussion of experiment 2

In experiment 2, we investigated the interactions between emoji and the semantic/grammatical structures of sentences, by asking the participants to converse while replacing at least one item in each sentence with emoji.

First, upon analysis of the position of the replacement emoji in the sentence (within, end, or whole), it was found that the rate of emoji within the sentence was higher than the chance level, with rates of emoji at the end of and as substitution for the whole sentences being lower. Compared with Cohn et al. (2019), similar results were obtained with regard to significantly higher occurrence rates of emoji within sentences and significantly lower rates at the end of and as substitution for whole sentences. Both these studies are consistent with the results of Potter et al.’s study (1986), in that when nouns or verbs in the middle of sentences tended to be replaced with images, they can be understood in almost the same way as sentences using only text. These results suggest that regardless of linguistic grammar, replacing words within sentences enables more easily comprehensible conversation.

In the grammatical category, the occurrence rate of emoji replacing nouns was significantly higher than the chance level, while those in other subcategories were significantly lower. Therefore, almost all the replacements were thought to be of nouns. Comparing these results with the experiments of Cohn et al. (2019) indicates that subject replacements are less common in Japanese speakers. Again, this contrastive finding can be ascribed to the fact that Japanese is a pro-drop language (Kato, 2012). In this sense, the frequency of emoji replacement in the grammatical category is influenced by participants’ linguistic grammar and its characteristics.

In the semantic category, objects were used at a rate significantly higher than the chance level. This is different from the results of Cohn et al. (2019), where places had the highest occurrence rate, but similarly actions were rarely replaced, with no major differences.

In multimodal relations, no category was replaced at a rate significantly higher than the chance level. The extratextual occurrence rate, in which the replacement is unrelated to the sentence meaning, was significantly lower. This is similar to the results of Cohn et al. (2019), suggesting a mutual interaction with semantic structure which makes sentence creation and understanding difficult in the case of replacement unrelated to the text.

In structural types, semantic lists were used at a frequency significantly higher than the chance level; responsive emotions were likewise frequently used. Temporal sequences were used at a significantly lower rate than the chance level, and the rate of unrelated lists was also low. This is also supported by the results of Cohn et al. (2019), showing that it is difficult to create or understand sentences where semantically unrelated emoji are frequently used in the categorical grammars or where replacement involves complicated sentences with temporal sequences.

## Overall discussion and conclusion

By following up the study by Cohn et al. (2019), this study examined whether there was a relationship between emoji usage patterns and native language grammar, specifically focusing on monolingual Japanese speakers and the Japanese language. The results of Experiment 1 suggested that conversation using emoji does not use universal common grammar regardless of language but is influenced by the language normally used. The results of Experiment 2 found that emoji replacement rarely affects subjects, influenced by the lesser importance of subjects in Japanese grammar. These results indicate that the use of emojis in conversation is influenced by the participants’ native language, particularly when the language does not directly participate in the message delivery. These findings corroborate Takano and Nakata’s (2013) study, demonstrating that the sequence of picture cards is influenced by the word order of the native language. This suggests that emojis and pictures, as visual modalities, operate similarly because they are static symbols and are treated akin to linguistic elements when used independently to convey a message. In addition, emojis are used in written language formats characterised by the linear arrangement of symbols. Because emojis evolve with this linearity, they may be strongly influenced by the word order of the language the user employs. In fact, Thamsen (2019) found that when several emojis are combined into a single analogue image, participants responded to it with shorter reaction times compared to linear emoji sequences. The linearity of emojis restricts the emoji’s potential capacity to convey rich and intricate expressions, similar to those found in written or visual languages.

Although hand gesture is also a visual modality, previous studies on silent gestures (e.g. Gershoff-Stowe & Goldin-Meadow, 2002; Goldin-Meadow et al., 2008; Langus & Nespor, 2010) showed that the gesture strings do not always follow the native language word order. This implies that the impact of native language grammar on visual modalities depends on their symbolic attributes. Specifically, it varies by their capacity to express meanings dynamically or statically, and whether a symbol's meaning is ambiguous or clear. For future research, it is crucial to explore how text and various type of visual modalities are integrated in message delivery.

The current study found that conversation using emoji may be influenced by the grammar and characteristics of the native language. This finding can be put to use towards smoother online communication. For example, texts and emojis can be typed more smoothly when emoji predictive text is matched to the user’s language and grammar of use. By considering visual modality such as emoji along with native languages, digital communication should become smoother.

The current study and the study by Cohn et al. (2019) used the same language for both members of the pair using emoji. Therefore, it has not yet been made clear in what order emoji will be used when users of different languages converse in pairs using emoji. The current study, in which the order of emoji was influenced by Japanese grammar, showed a high rate of SOV and OV patterns. However, it is possible that native Japanese speakers, when conversing with native English speakers, may use SVO word order for easier comprehension for the conversation partner. Further, the subjects frequently omitted in Japanese may be more clearly indicated in order to convey information. One future study can include an investigation of how emoji, a type of visual communication tool used around the world, are used in communication between people using different languages, in order to pursue emoji usage methods which promote smoother communication. As a future task, we will also examine if the finding from the current study is a robust phenomenon by increasing the number of participants, given that our sample size in this study was relatively small.

Another future task involves examining the extent to which the relationship with the conversation partner impacts text-emoji communication. As Pickering and Garrod (2006) stated, effective communication in dialogue is predicated on the alignment of similar representations between interlocutors. Studies on interaction have indeed found that interlocutors align their representations during dialogue at many different levels, such as the referential level (Cleland & Pickering, 2003), the syntactic level (Branigan et al., 2000), and even at the gesture level (Holler & Wilkin, 2011), with successful communication occurring when they become well aligned. In the current study, all pairs consisted of friends. Interactions between people who know each other, such as acquaintances or friends, may make communication more efficient than interactions between people who do not know each other, because acquaintances have more shared past experiences or vocabularies than strangers, allowing them to easily create alignments during the dialogue. Thus, it would be important to examine how the quality of the relationship between participants makes the conversation successful.

### Supplementary Information

Supplementary material 1.

## Data Availability

The datasets generated for the current study are available from the corresponding author upon reasonable request.

## References

[CR1] Boutet, I., LeBlanc, M., Chamberland, J. A., & Collin, C. A. (2021). Emojis influence emotional communication, social attributions, and information processing. *Computers in Human Behavior,**119*, 106722. 10.1016/j.chb.2021.106722 10.1016/j.chb.2021.106722

[CR2] Branigan, H. P., Pickering, M. J., & Cleland, A. A. (2000). Syntactic co-ordination in dialogue. *Cognition,**75*(2), 13–25. 10.1016/S0010-0277(99)00081-5 10.1016/S0010-0277(99)00081-5 10771277

[CR3] Cleland, A. A., & Pickering, M. J. (2003). The use of lexical and syntactic information in language production: Evidence from the priming of noun-phrase structure. *Journal of Memory and Language,**49*, 214–230. 10.1016/S0749-596X(03)00060-3

[CR4] Cohn, N. (2016). A multimodal parallel architecture: A cognitive framework for multimodal interactions. *Cognition,**146*, 304–323. 10.1016/j.cognition.2015.10.007 26491835 10.1016/j.cognition.2015.10.007

[CR5] Cohn, N., Engelen, J., & Schilperoord, J. (2019). The grammar of emoji? Constraints on communicative pictorial sequencing. *Cognitive Research: Principles and Implications,**4*(1), 1–18. 31471857 10.1186/s41235-019-0177-0 PMC6717234

[CR6] Gershoff-Stowe, L., & Goldin-Meadow, S. (2002). Is there a natural order for expressing semantic relations? *Cognitive Psychology,**45*, 375–412. 12480479 10.1016/S0010-0285(02)00502-9

[CR7] Goldin-Meadow, S., So, W. C., Özyürek, A., & Mylander, C. (2008). The natural order of events: How speakers of different languages represent events nonverbally. *Proceedings of the National Academy of Sciences,**105*(27), 9163–9168. 10.1073/pnas.0710060105 PMC2453738 18599445

[CR8] Hand, C. J., Burd, K., Oliver, A., & Robus, C. M. (2022). Interactions between text content and emoji types determine perceptions of both messages and senders. *Computers in Human Behavior Reports,**8*, 100242. 10.1016/j.chbr.2022.100242 10.1016/j.chbr.2022.100242

[CR9] Holler, J., & Wilkin, K. (2011). Co-speech gesture mimicry in the process of collaborative referring during face-to-face dialogue. *Journal of Nonverbal Behaviour,**35*, 133–153. 10.1007/s10919-011-0105-6

[CR10] Jackendoff, R., & Wittenberg, E. (2014). What you can say without syntax: A hierarchy of grammatical complexity. In F. Newmeyer & L. Preston (Eds.), *Measuring linguistic complexity* (pp. 65–82). Oxford University Press.

[CR11] Kato, K. (2012). Structural characteristics in Japanese sentences. *Bunka Gakuen University Bulletin Humanities/Social Science Research,**20*, 1–13.

[CR12] Kelly, R., & Watts, L. (2015). Characterising the inventive appropriation of emoji as relationally meaningful in mediated close personal relationships. In *Experiences of Technology Appropriation: Unanticipated Users, Usage, Circumstances, and Design*.

[CR13] Langus, A., & Nespor, M. (2010). Cognitive systems struggling for word order. *Cognitive Psychology,**60*(4), 291–318. 20189553 10.1016/j.cogpsych.2010.01.004

[CR14] Marno, H., Langus, A., Omidbeigi, M., Asaadi, S., Seyed-Allaei, S., & Nespor, M. (2015). A new perspective on word order preferences: The availability of a lexicon triggers the use of SVO word order. *Frontiers in Psychology,**6*(1183), 152231. 10.3389/fpsyg.2015.01183 PMC4534792 26321994

[CR15] Na'aman, N., Provenza, H., & Montoya, O. (2017). Varying linguistic purposes of emoji in Twitter context. In *Paper presented at the Proceedings of ACL 2017*, Student Research Workshop.

[CR16] Nagata, Y. (2020). Reiwajidai no yunikodo to ICT [Unicode and ICT in the Reiwa era]. *Fukuoka University Humanities Series,**52*(3), 807–837.

[CR17] Naito, D., & Yabuki, T. (2020). Nihongo to eigo ni okeru emoji no tukawarekata no bunsan hyougen ni motoduku hikaku [Comparison of how emoji are used in Japanese and English based on distributed representation]. In *The 82nd National Convention of Information Processing Society of Japan* (Vol. 1, pp. 455–456).

[CR18] Oyama, S. (2015). On the functions of the modern pictogram. *Kukugakuin Journal,**116*(2), 1–16.

[CR19] Paggio, P., & Tse, A. P. P. (2022). Are emoji processed like words? An eye-tracking study. *Cognitive Science,**46*(2), e13099. 10.1111/cogs.13099 35122294 10.1111/cogs.13099

[CR20] Pickering, M. J., & Garrod, S. (2006). Alignment as the basis for successful communication. *Research on Language and Computation,**4*(2), 203–228. 10.1007/s11168-006-9004-0 10.1007/s11168-006-9004-0

[CR21] Potter, M. C., Kroll, J. F., Yachzel, B., Carpenter, E., & Sherman, J. (1986). Pictures in sentences: Understanding without words. *Journal of Experimental Psychology: General,**115*(3), 281. 10.1037/0096-3445.115.3.281 2944988 10.1037/0096-3445.115.3.281

[CR22] Takahashi, N., Ueno, M., Hamada, Y., & Shouji, Y. (2021). Emotional communication from writers to readers on a message including emoji. *Journal of Japanese Society of Kansei Engineering,**21*(1), 135–142.

[CR23] Takano, Y., & Nakata Y. (2013). Influence of the native language in non-verbal communication. In *Proceedings of the 27th annual conference of the Japanese society for artificial intelligence* (pp. 2031in–2031in).

[CR24] Thamsen, L. (2019). *Imact of linearity on emoji sequences*. [Master’s thesis, Tilburg University]. http://arno.uvt.nl/show.cgi?fid=148608

[CR25] Yamaguchi, A., & Fujita K. (2019). Generating emoji with conditional variational autoencoders and word embedding. In *Proceedings of the 33rd annual conference of the Japanese society for artificial intelligence* (pp. 2L4J904–2L4J904).

