# Supplementary material for: Grammatical structures of emoji in Japanese-language text conversations
Source: Cogn Res Princ Implic. 2024 Jul 29;9:49. doi: 10.1186/s41235-024-00571-9 (PMC11286883; doi:10.1186/s41235-024-00571-9)
Supplement: Supplementary file 1 — Supplementary material 1. [file 41235_2024_571_MOESM1_ESM.docx]

Supplementary material

Table 1. Mean proportions, standard deviations, standard errors, and confidence intervals for each utterance type used in emoji-only conversations.

|  |  |  |  |  | 95% Confidence Intervals | |
| --- | --- | --- | --- | --- | --- | --- |
|  |  | Mean | SD | SE | Lower | Upper |
| One-unit grammars | Formulaic expressions | 0.14 | 0.09 | 0.02 | 0.10 | 0.18 |
|  | Responsive emotions | 0.24 | 0.17 | 0.04 | 0.16 | 0.32 |
|  | Single-word responses | 0.15 | 0.11 | 0.02 | 0.10 | 0.20 |
| Linear grammars | Temporal sequence | 0.03 | 0.06 | 0.01 | 0.00 | 0.06 |
|  | Unrelated list | 0.01 | 0.02 | 0.00 | 0.00 | 0.02 |
|  | Semantic list | 0.09 | 0.08 | 0.02 | 0.05 | 0.13 |
|  | Reduplication | 0.20 | 0.20 | 0.04 | 0.11 | 0.29 |
| Categorical grammars | S-V-O | 0.00 | 0.01 | 0.00 | 0.00 | 0.01 |
|  | S-O-V | 0.08 | 0.09 | 0.02 | 0.04 | 0.13 |
|  | O-S-V | 0.01 | 0.02 | 0.00 | 0.00 | 0.02 |
|  | O-V-S | 0.00 | 0.01 | 0.00 | 0.00 | 0.01 |
|  | V-O-S | 0.00 | 0.00 | 0.00 | --- | --- |
|  | V-S-O | 0.01 | 0.02 | 0.01 | -0.01 | 0.02 |
|  | S-V | 0.03 | 0.04 | 0.01 | 0.01 | 0.05 |
|  | S-O | 0.00 | 0.00 | 0.00 | --- | --- |
|  | O-S | 0.00 | 0.01 | 0.00 | 0.00 | 0.01 |
|  | O-V | 0.23 | 0.16 | 0.04 | 0.15 | 0.30 |
|  | V-S | 0.00 | 0.02 | 0.00 | 0.00 | 0.01 |
|  | V-O | 0.00 | 0.00 | 0.00 | --- | --- |
| Simple phrase grammars | Embedded | 0.06 | 0.09 | 0.02 | 0.01 | 0.10 |
| Other | Metonymy | 0.00 | 0.01 | 0.00 | 0.00 | 0.01 |
|  | Rebus | 0.00 | 0.00 | 0.00 | --- | --- |
|  | Whole image | 0.06 | 0.07 | 0.02 | 0.03 | 0.10 |
|  | Affixation | 0.04 | 0.06 | 0.01 | 0.02 | 0.07 |

|  |  |  |  | 95% Confidence Intervals | |
| --- | --- | --- | --- | --- | --- |
|  | Mean | SD | SE | Lower | Upper |
| Formulaic expressions | 0.27 | 0.12 | 0.03 | 0.22 | 0.33 |
| Responsive emotions | 0.44 | 0.19 | 0.04 | 0.35 | 0.53 |
| Single-word responses | 0.28 | 0.14 | 0.03 | 0.22 | 0.35 |

Table 2. Mean proportions, standard deviations, standard errors, and confidence intervals of the use of each subcategory in ‘one-unit grammars”.

Table 3. Mean proportions, standard deviations, standard errors, and confidence intervals of the use of each subcategory in ‘linear grammars’.

|  |  |  |  | 95% Confidence Intervals | |
| --- | --- | --- | --- | --- | --- |
|  | Mean | SD | SE | Lower | Upper |
| Temporal sequence | 0.08 | 0.14 | 0.03 | 0.02 | 0.15 |
| Unrelated list | 0.03 | 0.07 | 0.02 | 0.00 | 0.06 |
| Semantic list | 0.34 | 0.33 | 0.07 | 0.19 | 0.49 |
| Reduplication | 0.55 | 0.29 | 0.07 | 0.41 | 0.69 |

Table 4. Mean proportions, standard deviations, standard errors, and confidence intervals of the use of each subcategory in ‘categorical grammars’.

|  |  |  |  | 95% Confidence Intervals | |
| --- | --- | --- | --- | --- | --- |
|  | Mean | SD | SE | Lower | Upper |
| S-V-O | 0.02 | 0.05 | 0.01 | -0.01 | 0.04 |
| S-O-V | 0.21 | 0.22 | 0.05 | 0.11 | 0.31 |
| O-S-V | 0.03 | 0.08 | 0.02 | -0.01 | 0.07 |
| O-V-S | 0.00 | 0.02 | 0.00 | 0.00 | 0.01 |
| V-O-S | 0.00 | 0.00 | 0.00 | --- | --- |
| V-S-O | 0.01 | 0.04 | 0.01 | -0.01 | 0.03 |
| S-V | 0.10 | 0.15 | 0.03 | 0.03 | 0.17 |
| S-O | 0.00 | 0.00 | 0.00 | --- | --- |
| O-S | 0.00 | 0.02 | 0.00 | 0.00 | 0.01 |
| O-V | 0.61 | 0.26 | 0.06 | 0.49 | 0.73 |
| V-S | 0.02 | 0.07 | 0.02 | -0.02 | 0.05 |
| V-O | 0.00 | 0.00 | 0.00 | --- | --- |

Table 5. Mean proportions, standard deviations, standard errors, and confidence intervals for each semantic/grammatical type substituted by emoji.

|  |  |  |  |  | 95% Confidence Intervals | |
| --- | --- | --- | --- | --- | --- | --- |
|  |  | Mean | SD | SE | Lower | Upper |
| Grammatical categories | Subject | 0.01 | 0.02 | 0.00 | 0.00 | 0.02 |
|  | Object | 0.08 | 0.10 | 0.02 | 0.04 | 0.13 |
|  | Verb | 0.07 | 0.11 | 0.02 | 0.02 | 0.12 |
|  | Noun | 0.75 | 0.16 | 0.04 | 0.67 | 0.82 |
|  | Adjective | 0.06 | 0.10 | 0.02 | 0.01 | 0.10 |
|  | Adverb | 0.03 | 0.07 | 0.02 | 0.00 | 0.07 |
| Semantic categories | Semantic object | 0.42 | 0.16 | 0.04 | 0.34 | 0.49 |
|  | Animate object | 0.15 | 0.16 | 0.04 | 0.07 | 0.23 |
|  | Action | 0.10 | 0.11 | 0.02 | 0.04 | 0.15 |
|  | Property | 0.12 | 0.13 | 0.03 | 0.07 | 0.18 |
|  | Location | 0.21 | 0.17 | 0.04 | 0.13 | 0.29 |
| Multimodal relations | Extratextual | 0.10 | 0.13 | 0.03 | 0.04 | 0.16 |
|  | Associative | 0.31 | 0.31 | 0.07 | 0.16 | 0.46 |
|  | Metonymy | 0.38 | 0.33 | 0.07 | 0.23 | 0.53 |
|  | Redundant | 0.21 | 0.28 | 0.06 | 0.08 | 0.34 |
| Structural types | Temporal sequence | 0.01 | 0.04 | 0.01 | -0.01 | 0.03 |
|  | Unrelated list | 0.08 | 0.24 | 0.05 | -0.03 | 0.20 |
|  | Semantic list | 0.44 | 0.41 | 0.09 | 0.24 | 0.63 |
|  | Formulaic expressions | 0.09 | 0.15 | 0.03 | 0.02 | 0.16 |
|  | Responsive emotions | 0.28 | 0.33 | 0.07 | 0.13 | 0.43 |
|  | Reduplication | 0.10 | 0.17 | 0.04 | 0.02 | 0.18 |
